# Model-based inference from multiple dose, time course data reveals *Wolbachia* effects on infection profiles of type 1 dengue virus in *Aedes aegypti*

**DOI:** 10.1371/journal.pntd.0006339

**Published:** 2018-03-20

**Authors:** Caetano Souto-Maior, Gabriel Sylvestre, Fernando Braga Stehling Dias, M. Gabriela M. Gomes, Rafael Maciel-de-Freitas

**Affiliations:** 1 Instituto Gulbenkian de Ciência, Oeiras, Portugal; 2 Laboratório de Mosquitos Transmissores de Hematozoários, Instituto Oswaldo Cruz, Fiocruz, Rio de Janeiro, Brazil; 3 Gabinete da Presidência, Fiocruz, Rio de Janeiro, Brazil; 4 CIBIO-InBIo, Centro de Investigação em Biodiversidade e Recursos Genéticos, Universidade do Porto, Porto, Portugal; 5 Liverpool School of Tropical Medicine, Liverpool, United Kingdom; 6 Instituto Nacional de Ciência e Tecnologia em Entomologia Molecular (INCT-EM)/CNPq, Rio de Janeiro, Brazil; Johns Hopkins University, Bloomberg School of Public Health, UNITED STATES

## Abstract

Infection is a complex and dynamic process involving a population of invading microbes, the host and its responses, aimed at controlling the situation. Depending on the purpose and level of organization, infection at the organism level can be described by a process as simple as a coin toss, or as complex as a multi-factorial dynamic model; the former, for instance, may be adequate as a component of a population model, while the latter is necessary for a thorough description of the process beginning with a challenge with an infectious inoculum up to establishment or elimination of the pathogen. Experimental readouts in the laboratory are often static, snapshots of the process, assayed under some convenient experimental condition, and therefore cannot comprehensively describe the system. Different from the discrete treatment of infection in population models, or the descriptive summarized accounts of typical lab experiments, in this manuscript, infection is treated as a dynamic process dependent on the initial conditions of the infectious challenge, viral growth, and the host response along time. Here, experimental data is generated for multiple doses of type 1 dengue virus, and pathogen levels are recorded at different points in time for two populations of mosquitoes: either carrying endosymbiont bacteria *Wolbachia* or not. A dynamic microbe/host-response mathematical model is used to describe pathogen growth in the face of a host response like the immune system, and to infer model parameters for the two populations of insects, revealing a slight—but potentially important—protection conferred by the symbiont.

## Author summary

Infection is usually assayed as a static observation of a pathogen within a host; it is, nevertheless, a dynamic process that cannot be described from a single time point and arbitrary conditions. Results based on the usual methods are a snapshot of a convenient laboratory condition; a more comprehensive data set is required to describe the entire process of infection from inoculation of the host with a microorganism to establishment of a systemic infection, or elimination of the threat by the host. We design an experiment that takes into account increasing pathogen challenges to a mosquito host and viral levels along time; we use a dynamic mathematical model to analyze the resulting data set. The entire framework is used to compare susceptibility to dengue virus of *Aedes aegypti* mosquitoes either carrying the *Wolbachia* symbiont or not. Instead of a simple pairwise comparison, we are able to compare infection profiles and parameters associated to host immune processes in this insect-symbiont-virus system.

## Introduction

Infection is a complex and dynamic process that starts with a host coming in contact with pathogens, and ends with the latter either being eliminated by the former or becoming established inside it. A comprehensive analysis of host invasion by a microorganism requires a thorough description of the host biology, such as physical compartments and barriers, important tissues and organs, and immune responses, as well as the microbial processes, and the interaction of these many components [1]; missing parts in this description limit the thorough understanding of infection.

Due to constraints in time, resources, as well as analysis tools, any study must simplify its scope. Experimental assays typically rely on static measurements such as pathogen level at some specific time point, as well as restrictive laboratory conditions like a typical challenge dose [2]. While these traditional approaches can be useful to determine the effect of a large perturbation to the host-microbe system—such as gene knockouts or different pathogen strains—they are limited to a snapshot of the process under arbitrary conditions.

On the theoretical side, mathematical models of within-host pathogen dynamics are often disconnected from data, or use convenience data samples [3]. Because of that, the findings from modeling studies are only rarely comparable to those of more traditional experimental approaches.

Despite having lagged the establishment of population transmission—or between-host—mathematical models by several decades [4], quantitative descriptions of pathogen proliferation along time within-host have considerable history [5, 6]. Typically these take the form of a few coupled differential equations, and include simple constant-rate pathogen growth, with immune response-dependent pathogen death, and immunity described as either induced by the pathogen [7], constitutive [8], or both. Most models assume deterministic increase in pathogen load after entering the host, elimination being possible only by chance; less common are models that deterministically predict bistable outcomes in the form of either establishment or elimination [9].

Describing infection dynamically beyond a purely theoretical construct therefore requires specific time course data on the components described by the mathematical model; ideally, the data and its interpretations should also relate to the bulk of experimental research in host-pathogen infection experiments, and to the existing theoretical models available.

This work does not purport to, on its own, change and unify disparate fields, but instead it is a complete attempt at design, execution, and model-based analysis of a large experiment consisting of multiple initial dose challenges and time points that describe a range of progressions along time and outcomes of infection, from elimination to establishment. The data set produced illustrates how looking at any one dose and time point can only give a limited glimpse into the process of infection. Given the patterns visible in the data, a bistable model is found to be suitable to the broad features observed: it is able to describe increase in microbe levels up to establishment of systemic infection, as well as decrease and elimination of the pathogen.

*Aedes aegypti* mosquito hosts were infected with serotype 1 of dengue virus (DENV-1) previously circulating in the city of Rio de Janeiro, Brazil. The choice of the less tractable DENV-1 is a deliberate one, considering the small literature availability, and also the fact that experiments are mainly conducted with DENV-2, due to its ease of cultivation in C6/36 mosquito-cell culture. This bias further restricts knowledge about dengue more generally, and perpetuates many knowledge gaps in the difference between dengue serotypes. Besides using a system relevant to human health for a novel analysis, we infect a population of mosquitoes carrying a strain of the bacterium *Wolbachia*, a maternally inherited symbiont introduced as means of controlling dengue as well as Zika and chikungunya viruses [10, 11].

We fit the model to data of mosquito populations either carrying the symbiont *Wolbachia* or not, and compare the dynamic profiles and parameters estimated. Because the data set and model take into account both time and dose dimensions, the results are more general than those for typical laboratory conditions. In the light of the mathematical model and its inferred parameters, *Wolbachia* is shown to protect the mosquitoes from dengue virus infection, given the reduced time course profile of infection associated to increased recruiting and longer-lived host response.

We discuss how these results compare to past experiments and what they bring to future ones, the limitations we acknowledge in this particular work and how they can be overcome in future similar efforts, as well as the implications for the study of infection more generally and in other systems, particularly different dengue virus serotypes.

## Materials and methods

### Experiment design and execution

Around 1000 mosquito larvae were reared in plastic trays with approximately 1.5 liters of dechlorinated water and 0.9 g of Tetramin Tropical Flakes^®^ added every two days. Adult *Ae. aegypti* were maintained at 25 ± 3°C and relative humidity of 80 ± 5% for about 2-3 days for mating, with a sugar solution of 10% *ad libitum*. Two groups were used, *w*Mel*BR* (formed by backcrossing Brazilian wild males with *Wolbachia* infected Australian females, as showed on [12] and *w*Mel*TET* (obtained from treating *w*Mel*BR* mosquitoes with the antibiotic Tetracycline by three consecutive generations, healing *Wolbachia* in these insects). Thus, the two groups have strong genetic similarities regarding their background.

Dengue virus infection was performed with DENV-1 samples recently isolated from a patient in Rio de Janeiro and stored at −80°C. DENV-1 was initially amplified to a 10^8^ TCID_50_/mL and later passed through a ten-fold serial dilution, producing five different titers, from 10^8^ to 10^4^ TCID_50_/mL.

#### Intrathoracic infection with dengue virus

In order to obtain better precision on the initial DENV-1 level in *Ae. aegypti* mosquitoes, we controlled the volume and viral load received by each mosquito. To that end 207nL of solution of DENV-1 were artificially inoculated directly into each mosquito body cavity (haemocoel) through microinjections (Nanoject^®^ Drummond Scientific Company).

It is worth noting that when a mosquito is infected via intrathoracic inoculation, the viral particles are deposited directly in the mosquito haemocoel, bypassing the midgut barrier, but that there are nevertheless a number of further challenges for the pathogen to achieve systemic infection [1].

#### Virus detection and quantification

The detection of viral RNA in each individual was done by qRT-PCR [13]. Mosquitoes were kept under laboratory conditions. At three time points—3, 7 and 14 days after intrathoracic infection (d.p.i.)—they were individually stored in tubes and cryopreserved in a −80°C freezer for the preservation of viral RNA. After the collection and storing of all mosquitoes in all doses and time points, *Ae. aegypti* were individually assayed for viral load.

For RNA extraction each mosquito was, separately, put into a 2.0mL vial-tube with one glass bead 2mm and 50 *μ*L of PBS Buffer one-fold. The mosquitoes were then beaten for 90 seconds on Bead-beater machine (Biospec Products). After that, we performed the RNA extraction using High Pure Nucleic Acid kit (Roche) commercial kit following the manufacturer instructions. The RNAs were then quantified on Nanodrop Spectrophotometer (Thermo Scientific) and diluted to 50ng/*μ*L. For virus detection, we used a pair of primers DENV-Forw: 5’-AAG GAC TAG AGG TTA GAG GAG ACC C- 3’ and DENV-Rev: 5’- CGT TCT GTG CCT GGA ATG ATG- 3’ and DENV: 5’-HEX/AAC AGC ATA TTG ACG CTG GGA GAG ACC AGA/3BHQ_1/3’ probe that amplifies a 109pb fragment.

#### *Wolbachia* detection

Detection of *Wolbachia* was based on amplification of WD0513 gene. The following primers were used to amplify a fragment of 110bp: TM513-Forw: CAA ATT GCT CTT GTC CTG TGG and TM513-Rev: GGG TGT TAA GCA GAG TTA CGG, and the probe 5’-/FAM/ TGA AAT GGA AAA ATT GGC GAG GTG TAG G—3BHQ_1/3’. On the same reaction, a ribosomal gene from *Ae. aegypti* that amplifies a fragment of 68bp was analyzed with the following primers: RPS17-Forw: 5’- TCC GTG GTA TCT CCA TCA AGC T- 3’ and RPS-Rev: 5’- CAC TTC CGG CAC GTA GTT GTC- 3’. We also used the probe RPS17: 5’-/HEX/CAG GAG GAG GAA CGT GAG CGC AG/3BHQ_1/-3’.

We carried out two different mixes, the first one, a monoplex, to detect dengue virus and the second one, a duplex, to detect *Wolbachia* and *Ae. aegypti* genes. The qPCR reactions were performed in a total volume of 10 *μ*L, including 2.5 *μ*L (125 ng) of RNA, 2.5 *μ*L of TaqMan^®^ Fast Virus 1-Step Master Mix (Thermo Scientific), 0.5 *μ*L of each primer (reverse and forward) at concentration of 10 *μ*M and 0.25 *μ*L of each probe at 10 mM. Ultra-pure water was added to adjust the final volume to 10 *μ*L. In all 96-well plates, mosquito controls were used. Four wells were used with a pool of five *Ae. aegypti* mosquitoes each, two infected with *Wolbachia* and two with uninfected mosquitoes, all reared in insectary using the same protocol described in the methods. For dengue virus, we used in addition to a positive control, a standard curve in a serial dilution from 10^3^ to 10^8^. Additionally, a mock-infected control was used in all plates, as well as a negative control with no RNA added. All samples were analyzed on ViiA7 Real-Time PCR System (Thermo Scientific).

### Structure of the data set

The experimental design, therefore included 6 different challenge doses and 3 time points for both *Wolbachia*-carrying and *Wolbachia*-free groups. The numbers of mosquitoes assayed by qPCR for each of the 36 experimental conditions are shown in a supplementary Table A in S1 Text. For the purpose of computing statistical correlations between dengue and *Wolbachia* titers, only conditions with no fewer than four detectable pairs of data points were used to avoid obtaining artificially high correlations due to lack of data points. For inference purposes, conditions with six or fewer mosquitoes and non-zero titers were not used.

To produce the final data set used for all analyses hereafter the levels of DENV-1 were normalized by dividing by the mosquito Ribosomal Protein S17 (RPS) gene. The viral titers being relative to the mosquito gene, the lowest value in the data set was set to unity, and all others were adjusted accordingly and rounded to the closest integer values; this scaling neither affects the relative titers nor the subsequent analyses.

**Fig 1:**
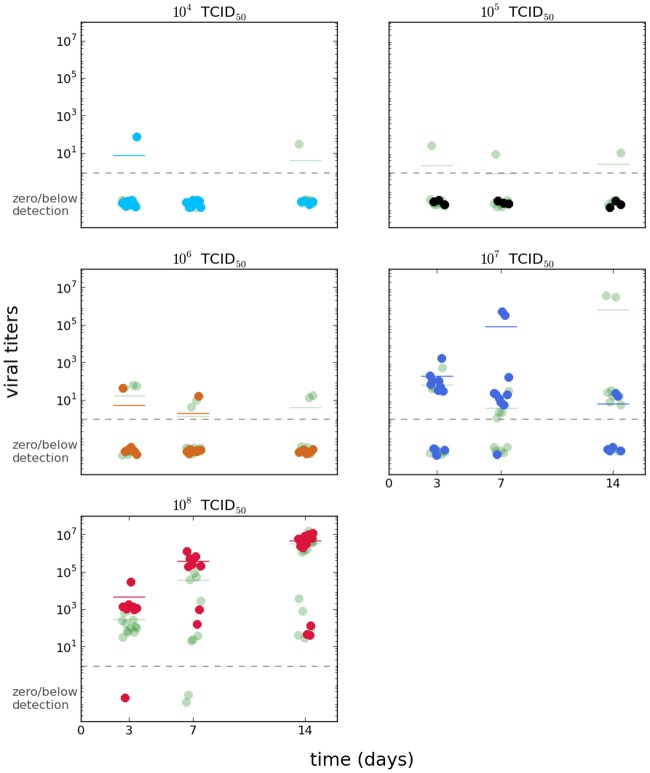
Viral levels in symbiont-free mosquitoes. DENV-1 viral titer data for *w*Mel*TET* group (colors) overlaid to that of the *w*Mel*BR* group (light green).

**Fig 2:**
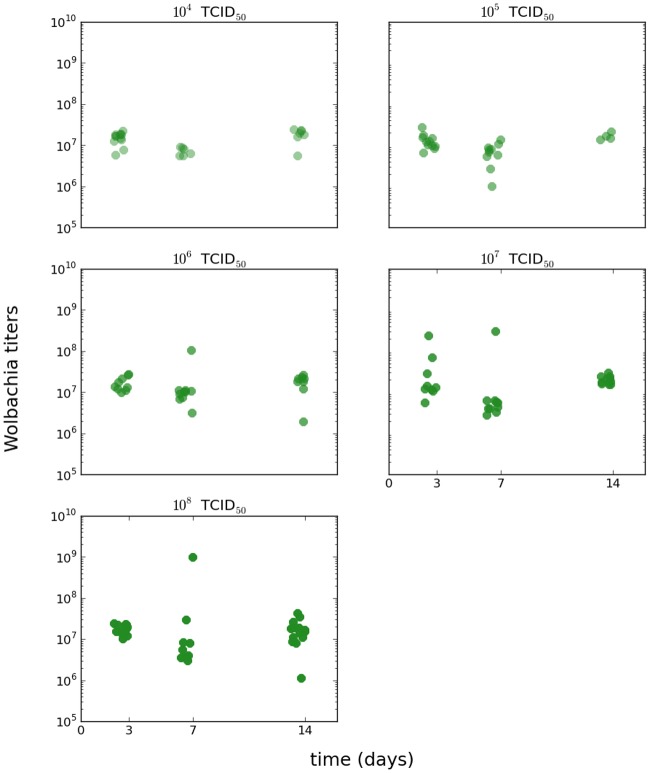
*Wolbachia* levels in symbiont-carrying mosquitoes. *w*Mel*BR* levels in symbiont-carrying population.

The data set for viral levels in the *w*Mel*TET* group is shown in the foreground of Fig 1, with the viral titers for the *w*Mel*BR* shown in light-shaded color for comparison. For ease of visualization, the data set for viral levels in the *w*Mel*BR* group is shown in supplementary Fig A in S1 Text with this group in the foreground instead. For the *w*Mel*BR* group, the symbiont levels were computed relative to the same house keeping gene used as a standard for the viral titer data; otherwise the procedure was the same as for the *w*Mel*TET* group. The *w*Mel*TET* group was used as a negative control, and every sample had undetectable qPCR levels of the symbiont, as expected. The *Wolbachia* levels are shown in Fig 2.

### Mathematical models

The mathematical model used in this work is a slightly modified version from the model proposed by Pujol et al. [9]. Most within-host models found in the literature describe microbe reproduction as a constant rate, resulting in exponential growth in the absence of any other process; most also treat microbe killing as an immunity-dependent process, resulting in non-linear terms with both immune response and microbe density interacting [4–8]. These features are also present in this model.

Other than that, descriptions often diverge in what is the origin of the immune response. Models may assume it is constitutive, induced, or both [4]. Because these descriptions generally do not refer to any specific immune pathway, and it is more of a mathematical construct, we henceforth describe this component of the model with the more generic term host response. These features are included in our model, which is described by two differential equations, one for the pathogens, one for host response (with terms for both constitutive and induced processes), as shown by the system of Eq 1.

$$\begin{array}{l} \frac{dP}{dt}=rP-\delta_{P}PR-kP^{2}\\ \frac{dR}{dt}=\alpha +\lambda P-\gamma R-\delta_{R}PR \end{array}$$ (1)

In this model *r* is the growth rate of pathogens; growth is self-limiting due to the negative quadratic term, −*kP*^2^. Additional decreases in pathogen numbers are governed by a non-linear term, −*δ*_*P*_*PR*, indicating an increased rate of destruction of pathogen units when there is a host response *R*—the intensity of this response is governed by its own differential equation.

The host response can be described by pathogen-independent components, *α*, a constant rate of recruitment of the response minus a linear death rate, −*γR*, as well as pathogen-dependent components, λ*P*, describing the rate of recruitment of the response in the presence of pathogens minus a non-linear term, −*δ*_*R*_*PR*, representing the pathogen-induced destruction, use, or wear of the host response.

The difference between this formulation and that of Pujol et al. [9] is a logistic-like growth profile induced by the quadratic term; in the absence of a host response, pathogens follow a logistic growth, growing initially at nearly exponential rate and saturating as the population reaches carrying capacity. The same is true for a small response incapable of eliminating the pathogen; in that case growth is a little slower but at high levels it is limited mainly by the quadratic decrease resulting in a stable level of pathogens and response, as opposed to unlimited growth [9]. The importance of this feature to explain specific features of our data set, as well as the implication for the possible mathematical solutions of the system are discussed in the results section.

### Statistics and inference

Correlations were computed between virus and *Wolbachia*, with the data stratified by dose and time as well as with the entire aggregated data set, to assess quantitative relationships between naturally changing symbiont levels and the observed viral levels.

Bayesian inference of the parameters for model (1) was performed using a Markov Chain Monte Carlo implementation in the Python programming language [14]. A poisson distribution of errors was used to compute the likelihood of the parameters given the data. Convergence was assessed by stability of the chains, and replicate chains were run to make sure the same approximate values were obtained regardless of starting point of the Markov chain [15]. *Burn-in* was performed by discarding the initial samples; the first half was assumed to be enough given the total lengths of the chains and trace of the likelihood values.

Besides the parameters described above, the initial condition parameter $P_{0}^{high}$ and the dilution “dosefold” parameter were also estimated—because each dose is diluted equally from the previous concentration, the two parameters define all initial conditions for all challenge doses by successively dividing the highest dose by the dilution value. Most parameters were kept fixed between the *w*Mel*TET* and *w*Mel*BR* groups, as described in the results section. The λ and *δ*_*R*_, as well as the initial condition *P*_0_ and dilution parameters are allowed to vary between the two groups. These choices are detailed in the discussion section.

Uniform priors were used on a wide range of positive values; a gamma-distributed prior is used for the dilution parameter, since it is known that a tenfold dilution was done for each lower dose. To reduce uncertainty, a gamma prior is also used for the growth and initial condition parameters; to that end a simple linear least-squares regression is computed for the highest dose logarithmic values, where the slope of straight line would give the exponential growth rate, and the intercept the initial condition—those values are used as the mean of the gamma distribution. In addition to the main analysis a generalized linear model is fitted to the data to assess significance of the variables in the experimental design (see supplementary material).

## Results

### Data description and linear statistical analyses

Broad patterns can be observed directly from Fig 1: the two lower doses have essentially zero infection, with only a couple of data points at very low levels. The intermediate dose has already a higher proportion of infected individuals, but still at low levels. The higher doses show a clear trend of increasing pathogen levels along time, with the exception of the second highest dose at the last time point (10^7^ TCID_50_); however, the unexpected pattern may be an effect of the low number of data points for that condition. Given our criteria for number of data points per condition described in the methods, this time/dose condition is not used for the model-based inference. In any case, in the next section we discuss more sophisticated methods that could be used in the future to deal with issues such as low number of data points for any one condition without any *ad hoc* treatment.

One very important feature of the data is a number of data points far from the mean, which could represent samples from a long-tailed or bimodal distribution. This is especially visible in later time points of the second highest dose, but also on the highest challenge dose. The feature could be explained by bistability in the viral dynamics, stochasticity, or the combination of both, and it is further discussed later under the model predictions with and without stochasticity.

**Fig 3:**
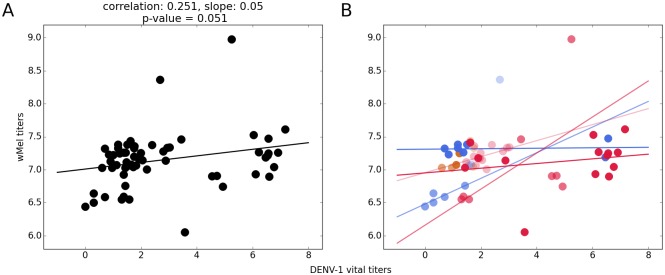
*Wolbachia*/DENV-1 level correlation. Correlations between DENV-1 and *Wolbachia* titers, displayed in log scale for the entire data set (A), and per-dose subsets (B)—color code follows that of the raw data.

Given that DENV and *w*Mel*BR* titers were measured for each individual mosquito, the correlation between virus and *Wolbachia* can be computed, and are shown in Fig 3 (separate panels are shown per dose and time point in Fig B in S1 Text). There is a significant (*p* = 0.05) but not high (*R*^2^ = 0.25) positive correlation for the whole data set.

We also compute the correlations for each time point and dose combination individually, as long as there were more than 3 non-zero data points in the condition tested. Some correlations were somewhat higher, but for the most part not significant at the 5% level, and were all positive. These are shown in Table 1.

**Table 1:** Virus/Symbiont correlation.

Dose (TCID_50_)	time (d.p.i.)	*R*^2^	*p*–value
10^7^	7	0.88	0.049
	14	0.1	0.82
10^8^	3	0.50	0.093
	7	0.57	0.14
	14	0.21	0.474

Given these results, *Wolbachia* is therefore treated not as a quantitative variable, but as a present/absent factor.

### Model solutions and forward simulation

There are three mathematical solutions to the system of differential Eq (1); the simplest being the pathogen-free solution ($P_{\text{free}}^{*}=0$), where constitutive recruitment and constant-rate elimination of immunity results in a stable equilibrium $R_{\text{free}}^{*}=\alpha /\gamma$. The other two solutions represent a stable establishment of pathogens, i.e. a systemic, persistent infection, and an unstable equilibrium that is of interest mainly to determine under which conditions the system will be tipped one way or the other. All three solutions are shown below:

$$\begin{array}{r} (\left(P_{\text{free}}^{*}=0;R_{\text{free}}^{*}=\frac{\alpha }{\gamma }\right),\\ (P_{\text{systemic}}^{*}=\frac{r\delta_{R}-\lambda \delta_{P}-\gamma k+\sqrt{{\left(\lambda \delta_{P}+\gamma k-r\delta_{R}\right)}^{2}-4k\delta_{R}(\alpha \delta_{P}-\gamma r)}}{2k\delta_{R}};\\ R_{\text{systemic}}^{*}=\frac{r\delta_{R}+\lambda \delta_{P}+\gamma k-\sqrt{{\left(\lambda \delta_{P}+\gamma k-r\delta_{R}\right)}^{2}-4k\delta_{R}(\alpha \delta_{P}-\gamma r)}}{2\delta_{P}\delta_{R}}),\\ (\hat{P}=\frac{r\delta_{R}-\lambda \delta_{P}-\gamma k-\sqrt{{\left(\lambda \delta_{P}+\gamma k-r\delta_{R}\right)}^{2}-4k\delta_{R}(\alpha \delta_{P}-\gamma r)}}{2k\delta_{R}};\\ \hat{R}=\frac{r\delta_{R}+\lambda \delta_{P}+\gamma k+\sqrt{{\left(\lambda \delta_{P}+\gamma k-r\delta_{R}\right)}^{2}-4k\delta_{R}(\alpha \delta_{P}-\gamma r)}}{2\delta_{P}\delta_{R}})) \end{array}$$

The bistability in the steady states of the model can produce the bimodal distribution of the later viral titers, observed in the data, as long as there are differences in the initial conditions of infection. Although the injected doses are controlled, it cannot be excluded that experimental or biological variation in the host or virus initial conditions explains observed bimodal outcomes in an otherwise adequate deterministic model. Alternatively, a stochastic implementation of the model acknowledges noise in the processes along the entire trajectory and gives a non-zero probability of observing a bimodal distribution even for identical initial conditions.

**Fig 4:**
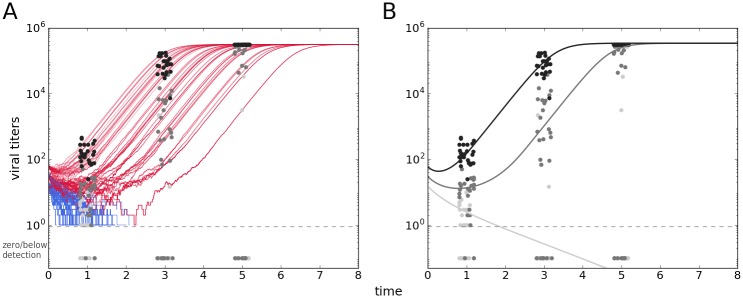
Model simulation results. Pseudo-data points sampled at different time points from a stochastic simulation, with the trajectories shown (A), and a deterministic simulation shown over the sample pseudo-data (B). Parameter values are: *r* = 3.5, *δ*_*P*_ = 0.042, *α* = 4.8, *γ* = 0.032, *δ*_*R*_ = 0.045, λ = 0.12, *k* = 10^−5^, *P*_0_ = 15, *dilution* = 2.

Numerical simulation of the system illustrates not only the equilibria, but the dynamic trajectory of the pathogens towards either establishment or elimination, and is shown in Fig 4. At selected time points the levels of pathogens can be sampled from the simulation, generating a pseudo-data set similar in structure to a real data set. With a somewhat arbitrary set of parameters it can be seen that some of the broad features of our real data could be reproduced by a stochastic simulation of the model (Fig 4A), and that some of it could be captured by a deterministic approximation (Fig 4B).

In the absence of the quadratic term the system has only one stable equilibrium, elimination of the pathogen (identical to the one shown above), and one unstable equilibrium [9]; if pathogens manage to grow beyond control of the response their growth is unbounded. For real data sets, pathogen levels are expected not to increase indefinitely, as is observed for our data set, therefore our attempt to explain the data set with this model does include the logistic-like term.

This forward approach gives us the output of the model given a set of parameters, but does not give anything beyond qualitative interpretations of any data set; in the next section we use the reverse approach with the *Aedes*-DENV-*Wolbachia* data set to infer the parameter values given our experimental data and compare the two populations of mosquitoes.

### Model-based bayesian inference

**Fig 5:**
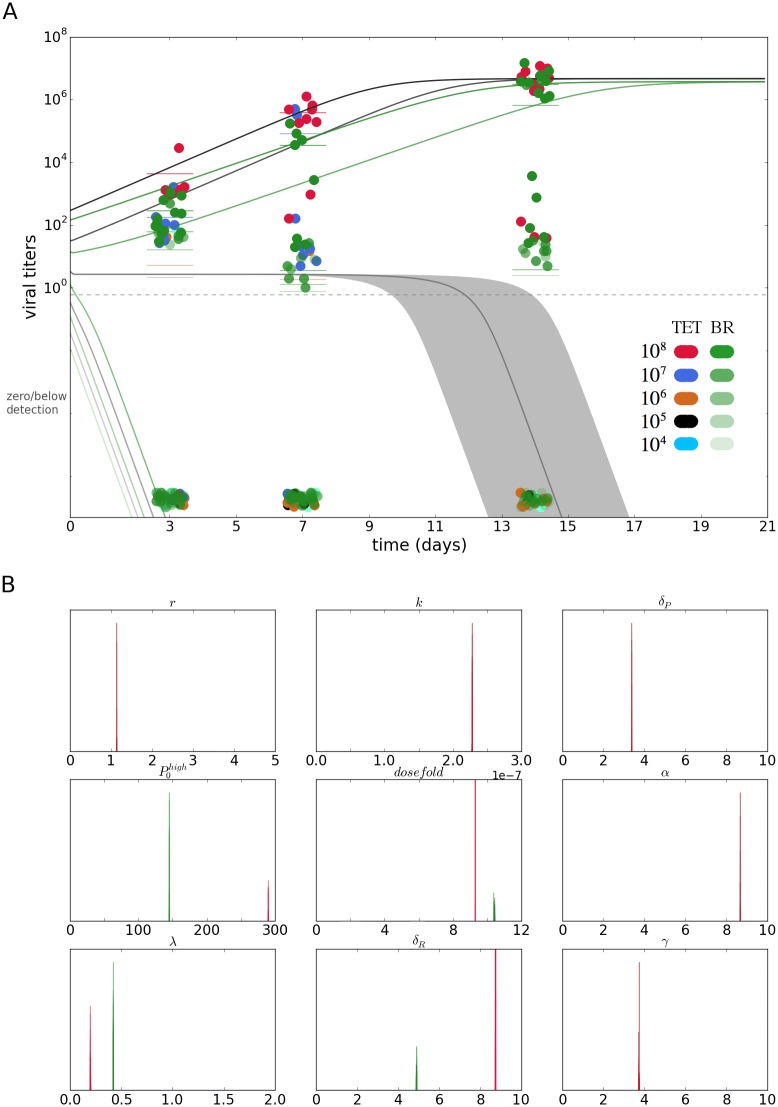
Dynamic model estimate and posterior parameter distributions. (A) Fit of dynamic host-pathogen model. Grayscale lines represent infection profiles from the *w*Mel*TET* group; green lines are the profiles for *w*Mel*BR* group. Green data points are the experimental data for the *w*Mel*BR* data, and the rest of the colors for the *TET group*. The dashed line indicates a visual separation of the detection limit, such that anything below it is effectively zero or undetectable. (B) Posterior distribution of estimated parameters. Where parameters are different between groups green color represents the posteriors for the *w*Mel-associated parameters.

The dynamic host-parasite model (1), described in the previous section, was fitted to the experimental data, as described previously. The results of the inference are shown in Fig 5; data points in the figure are a superposition of all panels in Fig 1, with the *w*Mel*TET* group data following the same color code, and the *w*Mel*BR* in green. The lines are the model prediction with the associated confidence intervals in a lighter shade.

The dynamic profile inferred (Fig 5A) shows not only lower initial pathogen levels, but generally lower titers along time for the population with *Wolbachia*. The highest two doses result in establishment of infection, although the increase is slower for the hosts carrying the symbiont. For the middle dose viruses are expected to persist at low levels and decay towards zero after around 10 days in the *w*Mel*TET* hosts, while in the *Wolbachia*-carrying hosts they drop below detection before day 1. The two lowest doses for both the *w*Mel*TET* and *w*Mel*BR* groups start from very low titers and rapidly decrease to even lower levels, making viral titers essentially zero for the entire time course.

Besides the dynamic profiles inferred, the values of individual parameters are shown in the lower panel (Fig 5B), breaking down which processes are responsible for the inferred dynamics. The initial condition parameter $P_{0}^{\left(high\right)}$ is greater for the *w*Mel*TET* group, and the dilution parameter is greater for the *w*Mel*BR* group, meaning estimated initial inoculum for the highest dose is lower for the latter group, and they are progressively smaller for lower doses—differences in the presumably equal initial inoculum are discussed in the next section. The additional parameters that differ between the two groups are λ, the pathogen-induced recruitment of the response, and *δ*_*R*_, the rate of destruction of the response once in contact with the pathogen. These parameters are greater and smaller, respectively, for the *Wolbachia*-carrying group; therefore, as interpreted by the model-based inference, the *w*Mel*BR* group recruits a response faster than the *w*Mel*TET* group, and this response wears out more slowly, i.e. persists longer once recruited.

## Discussion

Laboratory experiments are usually designed to maximize the success of infection by challenging susceptible mosquitoes with a single high viral dose, masking the range of possible outcomes of the host-pathogen interaction (in our system *Ae. aegypti* and DENV plus *Wolbachia*). Rather than using static readouts that only provide a snapshot of this process, the results shown here are a product of a multi-factorial experimental design. The interpretation of these results requires a quantitative framework that describes the initial infectious dose of the pathogen and considers the viral growth and the host response throughout time [9]. Performing a multiple-dose infectious challenge assay with 3 time points and 5 different DENV-1 dilutions on *Ae. aegypti* females—with the symbiont bacterium *Wolbachia* assumed to be a discrete variable that is either present or absent—revealed a mild although potentially important protection against the virus, conferred by the symbiont along time. To ascertain the importance of these findings, future experiments should emulate more natural viral challenge routes, and assess infection in specific host organs, such as the salivary glands given their role in transmission.

The intensity of pathogen transmission is largely influenced by the ability of mosquitoes to become infected by and transmit arboviruses. *Ae. aegypti* traits that are components of this vectorial capacity, such as susceptibility to DENV and the duration of the extrinsic incubation period (EIP), are likely to produce significant effects on the epidemiological trend during outbreaks [16, 17]. Therefore, a thorough quantitative description of the process is necessary to assess the impact of interventions on the vector population, and nevertheless excessive experimental simplification may preclude a useful understanding of the system beyond qualitative assessments or outright speculation.

The process of infection is extremely structured. Soon after the ingestion of an infected blood meal from a vertebrate host, viral particles are detected on *Ae. aegypti* midgut, where digestion takes place [18–20]. It is thought that some arboviruses can induce the activity of particular proteases to help disassemble the basal lamina surrounding the midgut and corollary disseminate to mosquito haemocoel [21]. The midgut infection barrier is an important bottleneck of viral replication inside the host [1]; by performing mosquito infection using intrathoracic injections, we allowed DENV to bypass it entirely, so that the control of viral load is due to other components of the host response, immunological or otherwise. Therefore, a component of vectorial competence is artificially removed in this experiment; extrapolations based on our data must take that into consideration because it should not fully represent a natural route of infection [22].

It is worth noting that a more natural oral infection should also result in an observable bistable pattern if an appropriate range of doses is used; however, the variation associated to feeding infected blood to mosquitoes, even in controlled laboratory conditions, as well as the additional biological steps to a systemic infection would lead to increased variation that may obscure the observed bistability.

Bypassing the midgut allows greater control of the pathogen dose received by each mosquito, and clearly shows the possibility of two opposite outcomes of either elimination of the pathogen, clearly seen for lower dilutions (10^4^ and 10^5^), or establishment, observed when the females were inoculated with the higher doses (10^7^ and 10^8^ TCID_50_).

Intuitively, it is expected that DENV dynamics inside an *Ae. aegypti* host is dependent on the initial amount of viral particles the mosquito has ingested during the infective blood meal. From the raw data at the assayed data points it can be seen that *w*Mel*BR* mosquitoes infected with high DENV doses generally have lower pathogen loads at specific time points, supporting the observation that *Wolbachia* has a protective effect. The viral growth can also be empirically observed to be slower in the *w*Mel*BR* group, which is supported by the model-inferred lower profile of pathogen levels in that group. On the other hand, the effect of *Wolbachia* on infection was less pronounced on the two lowest DENV doses since the virus titers rapidly decreased and remained close to zero for the entire time course for both groups.

Stochasticity is likely to play an important role on the observed infection outcomes. Duneau et al. [23] observe variation in bacterial levels along the course of infection, and interpret a bimodality in infection outcome under a two-piece model where each of the two outcome groups is assigned different trajectories after some time when the host can still control infection. While the model described by the authors is also deterministic, variation in the initial phase of infection is found to predict which hosts are able to reach a tipping point that would determine whether an increasing or decreasing function describes infection levels from them on. While bacteria and viruses will have many specificities to their invasion of a host, some broad features of the data observed by Duneau et al. [23] may be shared between different types of pathogens. We too interpret that variation in the initial phase, or conditions of infection may determine the final outcome, although under our model bistability is an emerging property of the host-pathogen interactions. Stochastic versions of this and other models will be able to further accommodate unexplained variation, as shown by our forward simulations, and incorporating it into inference could significantly improve model-based analyses [24].

That the observed effect of *Wolbachia* depends on the dose does not mean that it cannot be explained by a single, unified model. Dose-response models, for instance, can explain the expected proportion for a binary (infected/uninfected) outcome as the effect of a single susceptibility distribution [25]; similarly, the model proposed here explains viral levels along time and ultimately opposite outcomes as a function of dose and time for a single set of parameters. As inferred by the model, the observed differences are a consequence of differences in the initial pathogen loads and host responses. The predictions about host response parameters are themselves hypotheses, both qualitative and quantitative, which can be compared to observations of the host immune response like the expression of specific pathways [26]. This is an interesting future perspective that has nevertheless not been explored in this work.

Reproducing all the complexity of the real world in a controlled laboratory study is unrealistic, but we do make the distinction between not considering discrete categorical variables, as opposed to fixing variables in a continuous scale [27]. An example of the former is a single viral serotype or genotype which can cause an epidemic alone, and a factor like *Wolbachia* is normally absent in the mosquito population. On the other hand mosquitoes will invariably ingest blood from infected hosts with a potentially great range of DENV titers [28], artificially fixing it is an example of the latter. This study aims at incorporating variation in quantitative dimensions that are inescapable in any infection, although it does not exhaust all continuous or otherwise extensive number of variables that are likely to be important in the process. Notably, mosquito-virus interactions are related to the heterogeneity of DENV (serotypes, and at a finer scale, genotypes) and mosquito populations with their genetic background and environmental factors [29, 30]; these are not explored beyond the inbred population used for this study.

The model-based inferred DENV-1 infection profiles reveal otherwise obscure differences between *Ae. aegypti* mosquitoes and their *Wolbachia*-carrying counterparts, and the underlying parameters that determine the difference. These results corroborate the reported effect of *Wolbachia* [10] in a broad sense, but are more comprehensive, and include observed and/or predicted differences for specific conditions of previous studies. Therefore, previous results can only be interpreted as particular cases of this study, not the other way around. Analogously, the effect of other factors, when assessed, should be interpreted under a complete picture of their infection profiles, as opposed to looking at a single time point or challenge dose. Ignoring these dimensions could be especially counterproductive when comparing susceptibility to or infectivity of discrete serotypes, for instance.

The work presented here shows the combination of a dynamic model with a multiple dose challenge design that allows interpretation of a comprehensive data set beyond discrete factors or pairwise comparisons, and allows more concrete hypotheses about the biology to be tested. Improvement of the mathematical and statistical framework, as well as inclusion of more detailed biological processes in the model description could further refine the interpretation of infection.

## Supporting information

S1 Text Supplementary file containing the table of mosquitoes challenged and assayed, generalized linear model analysis, an alternate version of viral titer data figure, and the correlations plotted in separate panels. (PDF)

## References

[1] Franz A., Kantor A., Passarelli A. & Clem R. 2015 Tissue Barriers to Arbovirus Infection in Mosquitoes. Viruses 2015;7:3741–3767. doi: 10.3390/v7072795 26184281 10.3390/v7072795 PMC4517124

[2] Gomes MGM, Lipsitch M, Wargo AR, Kurath G, Rebelo C, Medley GF, et al A Missing Dimension in Measures of Vaccination Impacts. PLOS Pathog. 2014;10:e1003849 doi: 10.1371/journal.ppat.1003849 24603721 10.1371/journal.ppat.1003849 PMC3946326

[3] Clapham HE, Tricou V, Van Vinh Chau N, Simmons CP, Ferguson NM. Within-host viral dynamics of dengue serotype 1 infection. J R Soc Interface. 2014;11:20140094 doi: 10.1098/rsif.2014.0094 24829280 10.1098/rsif.2014.0094 PMC4032531

[4] Alizon S, van Baalen M. Acute or Chronic? Within-Host Models with Immune Dynamics, Infection Outcome, and Parasite Evolution. The American Naturalist. 2008;172:E244–E256. doi: 10.1086/592404 18999939 10.1086/592404

[5] Sasaki A, Iwasa Y. Optimal growth schedule of pathogens within a host: Switching between lytic and latent cycles. Theoretical population biology. Academic Press; 1991;39:201–239. doi: 10.1016/0040-5809(91)90036-F 10.1016/0040-5809(91)90036-f 2057912

[6] Antia R, Koella JC, Perrot V. Models of the Within-Host Dynamics of Persistent Mycobacterial Infections. Proc R Soc B. 1996;263:257–263. doi: 10.1098/rspb.1996.0040 8920248 10.1098/rspb.1996.0040

[7] Antia R, Lipsitch M. Mathematical models of parasite responses to host immune defences. Parasitology. 1997;115:SupplS155–167. doi: 10.1017/S003118209700200X 10.1017/s003118209700200x 9571700

[8] Hamilton R, Siva-Jothy M, Boots M. Two arms are better than one: parasite variation leads to combined inducible and constitutive innate immune responses. Proc R Soc B. 2008;275:937–945. doi: 10.1098/rspb.2007.1574 18230594 10.1098/rspb.2007.1574 PMC2599937

[9] Pujol JM, Eisenberg JE, Haas CN, Koopman JS. The Effect of Ongoing Exposure Dynamics in Dose Response Relationships. PLOS Comput Biol. 2009;5:e1000399–12. doi: 10.1371/journal.pcbi.1000399 19503605 10.1371/journal.pcbi.1000399 PMC2685010

[10] Moreira LA, Iturbe-Ormaetxe I, Jeffery JA, Lu G, Pyke AT, Hedges LM, et al A *Wolbachia* Symbiont in *Aedes aegypti* Limits Infection with Dengue, Chikungunya, and *Plasmodium*. Cell. 2009;139:1268–1278. doi: 10.1016/j.cell.2009.11.042 20064373 10.1016/j.cell.2009.11.042

[11] Dutra HLC, Rocha MN, Dias FBS, Mansur SB, Caragata EP, Moreira LA. *Wolbachia* Blocks Currently Circulating Zika Virus Isolates in Brazilian *Aedes aegypti* Mosquitoes. Cell Host and Microbe. 2016;19:771–774. doi: 10.1016/j.chom.2016.04.021 27156023 10.1016/j.chom.2016.04.021 PMC4906366

[12] Dutra HLC, Santos dos LMB, Caragata EP, Silva JBL, Villela DAM, Maciel-de-Freitas R, et al From Lab to Field: The Influence of Urban Landscapes on the Invasive Potential of *Wolbachia* in Brazilian *Aedes aegypti* Mosquitoes. PLOS Negl Trop Dis. 2015;9:e0003689 doi: 10.1371/journal.pntd.0003689 25905888 10.1371/journal.pntd.0003689 PMC4408005

[13] Maciel-de-Freitas R, Koella JC, Lourenço-de-Oliveira R. Lower survival rate, longevity and fecundity of *Aedes aegypti* (Diptera: Culicidae) females orally challenged with dengue virus serotype 2. Trans R Soc Trop Med Hyg. 2011;105:452–458. doi: 10.1016/j.trstmh.2011.05.006 21700303 10.1016/j.trstmh.2011.05.006

[14] Patil A, Huard D, Fonnesbeck CJ. PyMC: Bayesian Stochastic Modelling in Python. J Stat Softw. 2010;35:1–81. doi: 10.18637/jss.v035.i04 21603108 PMC3097064

[15] Gelman A, Carlin JB, Stern HS, Rubin DB. Bayesian data analysis. CRC Press 2014.

[16] Massad E., Coutinho F. A. B. Vectorial capacity, basic reproduction number, force of infection and all that: formal notation to complete and adjust their classical concepts and equations. Mem. Inst. Oswaldo Cruz 2012;107,564–567. doi: 10.1590/S0074-02762012000400022 22666873 10.1590/s0074-02762012000400022

[17] Kuno G. Review of the Factors Modulating Dengue Transmission. Epidemiol Rev. 1995;17,321–335. doi: 10.1093/oxfordjournals.epirev.a036196 8654514 10.1093/oxfordjournals.epirev.a036196

[18] Woodring JL, Higgs S, Beaty BJ. Natural cycles of vector-borne pathogens 51-72 In: Beaty BJ, Marquardt WC. The biology of disease vectors. University Press of Colorado 1996

[19] Yazi-Mendoza MY, Salas-Benito JS, Lanz-Mendoza H, Hernández-Martínez S, del Angel RM. A putative receptor for dengue virus in mosquito tissues: localization of a 45-kDa glycoprotein. Am J Trop Med Hyg. 2002;67:76–84. doi: 10.4269/ajtmh.2002.67.76 12363068 10.4269/ajtmh.2002.67.76

[20] Salazar MI, Richardson JH, Sánchez-Vargas I, Olson KE, Beaty BJ. Dengue virus type 2: replication and tropisms in orally infected *Aedes aegypti* mosquitoes. BMC Microbiol. 2007;7:9 doi: 10.1186/1471-2180-7-9 17263893 10.1186/1471-2180-7-9 PMC1797809

[21] Means JC, Passarelli AL. Viral fibroblast growth factor, matrix metalloproteases, and caspases are associated with enhancing systemic infection by baculoviruses. Proc Natl Acad Sci USA; 2010;107:9825–9830. doi: 10.1073/pnas.0913582107 20457917 10.1073/pnas.0913582107 PMC2906863

[22] Lambrechts L, Scott TW Mode of transmission and the evolution of arbovirus virulence in mosquito vectors. Proc R Soc B. 2009;276:1369–1378. doi: 10.1098/rspb.2008.1709 19141420 10.1098/rspb.2008.1709 PMC2660968

[23] Duneau D, Ferdy JB, Revah J, Kondolf H, Ortiz GA, Lazzaro BP, Buchon N Stochastic variation in the initial phase of bacterial infection predicts the probability of survival in D. melanogaster. eLife. 2017;6:e28298 doi: 10.7554/eLife.28298 29022878 10.7554/eLife.28298 PMC5703640

[24] Andrieu C., Doucet A., Holenstein R. Particle Markov chain Monte Carlo methods. J R Stat Soc Series B. 2010;72,269–342. doi: 10.1111/j.1467-9868.2009.00736.x

[25] Pessoa D, Souto-Maior C, Gjini E, Lopes JS, Ceña B, Codeço CT, et al Unveiling Time in Dose-Response Models to Infer Host Susceptibility to Pathogens. PLOS Comput Biol. 2014;10:e1003773–9. doi: 10.1371/journal.pcbi.1003773 25121762 10.1371/journal.pcbi.1003773 PMC4133050

[26] Castillo JC, Shokal U, Eleftherianos I. Immune gene transcription in Drosophila adult flies infected by entomopathogenic nematodes and their mutualistic bacteria. J Insect Physiol. 2013;59:179–185. doi: 10.1016/j.jinsphys.2012.08.003 22902989 10.1016/j.jinsphys.2012.08.003

[27] Cohuet A, Osta MA, Morlais I, Awono-Ambene PH, Michel K, Simard F, et al *Anopheles* and *Plasmodium*: from laboratory models to natural systems in the field. EMBO Rep. 2006;7: 1285–1289. doi: 10.1038/sj.embor.7400831 17099691 10.1038/sj.embor.7400831 PMC1794687

[28] Ferguson NM, Kien DTH, Clapham H, Aguas R, Trung VT, Chau TNB, et al Modeling the impact on virus transmission of *Wolbachia*-mediated blocking of dengue virus infection of *Aedes aegypti*. Sci Transl Med. 2015;7:279ra37–279ra37. doi: 10.1126/scitranslmed.3010370 25787763 10.1126/scitranslmed.3010370 PMC4390297

[29] Rašić G, Schama R, Powell R, Maciel-de-Freitas R, Endersby-Harshman NM, Filipović I, et al Contrasting genetic structure between mitochondrial and nuclear markers in the dengue fever mosquito from Rio de Janeiro: implications for vector control. Evol Appl. 2015;8:901–915. doi: 10.1111/eva.12301 26495042 10.1111/eva.12301 PMC4610386

[30] Monteiro FA, Shama R, Martins AJ, Gloria-Soria A, Brown JE, Powell JR. Genetic Diversity of Brazilian *Aedes aegypti*: Patterns following an Eradication Program. PLoS Negl Trop Dis. 2014;8:e3167 doi: 10.1371/journal.pntd.0003167 25233218 10.1371/journal.pntd.0003167 PMC4169244

