# Supplementary material for: Model-based inference from multiple dose, time course data reveals Wolbachia effects on infection profiles of type 1 dengue virus in Aedes aegypti
Source: PLoS Negl Trop Dis. 2018 Mar 20;12(3):e0006339. doi: 10.1371/journal.pntd.0006339 (PMC5877886; doi:10.1371/journal.pntd.0006339)
Supplement: S1 Text — (PDF) [file pntd.0006339.s001.pdf]

Supplementary Material: Model-based Inference From Multiple Dose, Time Course Data Reveals *Wolbachia* Effects on Infection Profiles of Type 1 Dengue virus in *Aedes aegypti.*

Caetano Souto-Maior[1◑¤*], Gabriel Sylvestre[2◑], Fernando Braga Stehling Dias[3], M. Gabriela M. Gomes[4,5] Rafael Maciel-de-Freitas[2]

1 Instituto Gulbenkian de Ciência, Oeiras, Portugal
2 Laboratório de Mosquitos Transmissores de Hematozoários, Instituto Oswaldo Cruz, Fiocruz, Rio de Janeiro, Brazil
3 Gabinete da Presidência, Fiocruz, Rio de Janeiro, Brazil
4 CIBIO-InBIo, Centro de Investigação em Biodiversidade e Recursos Genéticos, Universidade do Porto, Porto, Portugal
5 Liverpool School of Tropical Medicine, Liverpool, United Kingdom.

◑These authors contributed equally to this work.
¤current address: National Heart, Lung, and Blood Institute, Bethesda, MD, USA
* correspondence: caetanosoutomaior@protonmail.com

Mosquito numbers and subgrouping data tables

A total of 616 mosquitoes were divided by symbiont status (absent, *w*Mel*TET* colony, and present *w*Mel*BR*) into two groups of approximately half that size, and challenged with five different *DENV-1* doses plus mock-infected controls injected with culture medium. These numbers are shown in table A.

Table A. Number of mosquitoes challenged and assayed for viral titers

		**dose ($TCID_{50}$/ml)**						
	**time point**	**10^4**	**10^5**	**10^6**	**10^7**	**10^8**	**ctrl**	**total**
*w*Mel*TET* challenged	**all**	**54**	**51**	**55**	**49**	**57**	**38**	**304**
*w*Mel*TET* qPCR assayed	3 d.p.i.	9	3	8	12	8	8	48
	7 d.p.i.	12	3	8	10	9	1	43
	14 d.p.i.	4	3	7	6	14	3	37
	**all (row sum)**	**25**	**9**	**23**	**28**	**31**	**12**	**128**
*w*Mel*BR* challenged	**all**	**51**	**57**	**55**	**54**	**55**	**40**	**312**
*w*Mel*BR* qPCR assayed	3 d.p.i.	12	11	9	9	13	11	65
	7 d.p.i.	6	10	10	10	10	6	52
	14 d.p.i.	7	4	8	10	14	6	49
	**all (row sum)**	**25**	**25**	**27**	**29**	**37**	**23**	**166**

Linear regression for factor effects

We use a generalized linear model as a baseline for significance of the factors included in the experimental design: dose, time, and presence or absence of the *Wolbachia* symbiont. The model is of the following form:

$$log\mu = dose + time + symbiont \quad (1)$$

errors are assumed to follow a Poisson distribution, like the main model, and significances of the factors were computed with a likelihood ratio test between the model shown above, and a model that does not include any of the terms for which the significance is being tested (e.g. $log\mu = dose + time$ is used as a reduced model to test significance of symbiont presence). All factors showed high significance ($p < 0.001$) under this linear model.

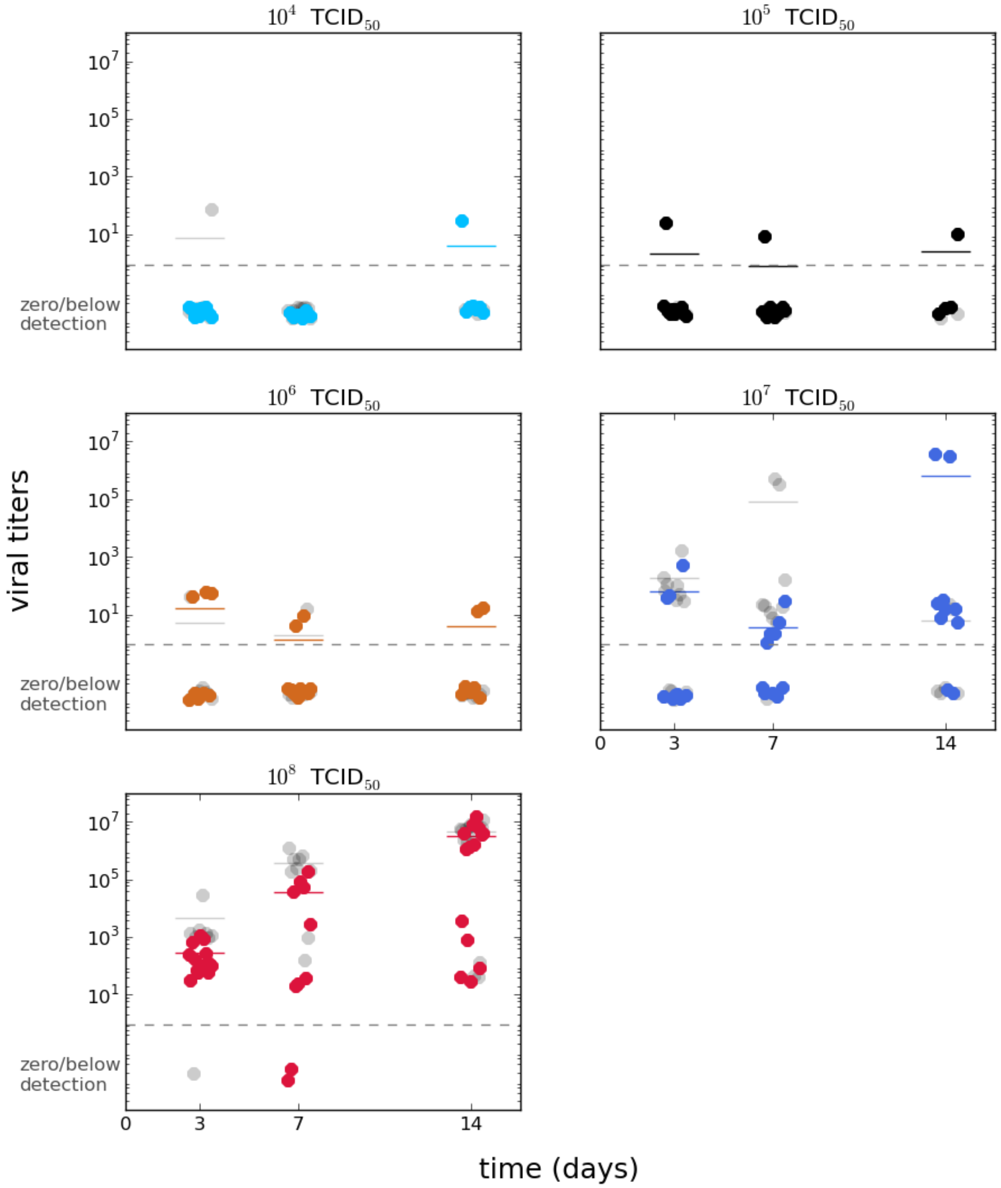

Fig A. Viral levels in symbiont-carrying mosquitoes. DENV-1 viral titer data for wMelBR group (colors) overlaid to that of the wMelTET group (light gray)

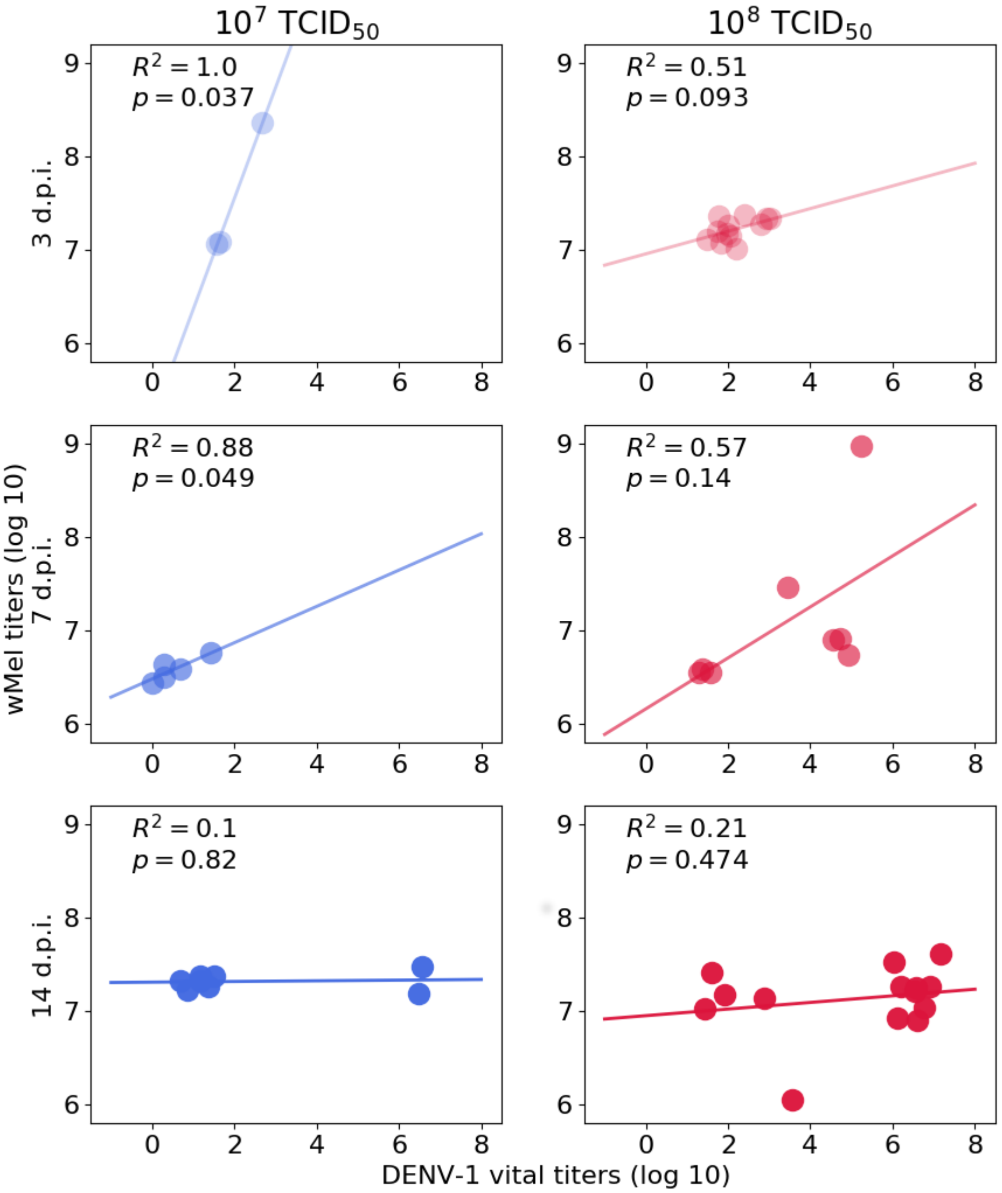

Fig B. *Wolbachia*/DENV-1 level correlation. Correlations between DENV-1 and *Wolbachia* titers displayed as per-dose subsets in separate panels for each dose and time point – color code follows that of the raw data.
